# Rosetting Responses of *Plasmodium*-infected Erythrocytes to Antimalarials

**DOI:** 10.4269/ajtmh.21-1229

**Published:** 2022-04-11

**Authors:** Wenn-Chyau Lee, Bruce Russell, Yee-Ling Lau, Francois Nosten, Laurent Rénia

**Affiliations:** ^1^ Department of Parasitology, Faculty of Medicine, Universiti Malaya, Kuala Lumpur, Malaysia;; ^2^ Infectious Diseases Labs (ID Labs), Agency for Science, Technology and Research (A*STAR), Singapore;; ^3^ Department of Microbiology and Immunology, University of Otago, Dunedin, Otago, New Zealand;; ^4^ Shoklo Malaria Research Unit, Mahidol-Oxford Tropical Medical Research Unit, Faculty of Tropical Medicine, Mahidol University, Mae Sot, Tak, Thailand;; ^5^ Nuffield Department of Medicine, University of Oxford, United Kingdom;; ^6^ Lee Kong Chian School of Medicine, Nanyang Technological University, Singapore;; ^7^ School of Biological Sciences, Nanyang Technological University, Singapore

## Abstract

In malaria, rosetting is a phenomenon involving the cytoadherence of uninfected erythrocytes to infected erythrocytes (IRBC) harboring the late erythrocytic stage of *Plasmodium* spp. Recently, artesunate-stimulated rosetting has been demonstrated to confer a survival advantage to *P. falciparum* late-stage IRBC. This study investigated the rosetting response of *P. falciparum* and *P. vivax* clinical isolates to ex vivo antimalarial treatments. Brief exposure of IRBC to chloroquine, mefloquine, amodiaquine, quinine, and lumefantrine increased the rosetting rates of *P. falciparum* and *P. vivax*. Furthermore, the ex vivo combination of artesunate with mefloquine and piperaquine also resulted in increased the rosetting rates. Drug-mediated rosette-stimulation has important implications for the therapeutic failure of rapidly cleared drugs such as artesunate. However, further work is needed to establish the ramifications of increased rosetting rates by drugs with longer half-lifves, such as chloroquine, mefloquine, and piperaquine.

In malaria, the rosetting phenomenon refers to a cytoadherence event in which a *Plasmodium* late-stage (trophozoite–schizont)-infected erythrocyte (IRBC) stably adheres to several uninfected erythrocytes (URBC).^1^ Rosettes protect the IRBC from phagocytosis.^2^^,^^3^ Recently, we reported that the late-stage IRBC of artesunate (AS; an artemisinin [ART] derivative)-resistant *P. falciparum*, rapidly formed more rosettes upon AS exposure, which conferred a survival advantage to the late stages, particularly the schizonts.^4^ Here, the effects of brief exposure of several commonly used antimalarials on the rosetting machinery of clinical isolates from the northwestern region of Thailand are reported.

*Plasmodium* spp.–infected blood samples were collected in the northwestern part of Thailand by Shoklo Malaria Research Unit under ethical guidelines: OxTREC 04-10 (University of Oxford) and TMEC 09-082 (Mahidol University). Adult participants provided informed written consent, whereas for each juvenile participant, a guardian provided informed written consent on his or her behalf. Information on the reagents and tools used is available in Supplemental Table 1. Experiments were conducted on ex vivo matured-parasite suspensions with ≥ 70% of the parasite population at late stages. Drug incubation was conducted with parasite suspension of 2% hematocrit, 1% parasitemia, in 20% human AB serum-enriched RPMI 1640 medium under in vitro cultivation conditions (37°C, > 90% humidity, gas mixture of 5% CO_2_, 5% O_2_, 90% N_2_) for 1 hour (defined as “brief drug exposure”). Cryopreserved clinical isolates were thawed using the 12% sodium chloride method.^5^ Rosetting assay was conducted using the Giemsa-wet mount method.^6^^,^^7^

The concentration range of drug compounds was set according to the geometric means of IC_50_ for the *P. falciparum* clinical isolates from Thailand.^8^^–^^12^ Chloroquine (CQ) and amodiaquine (AMQ) were dissolved in double distilled water. Mefloquine (MQ), AS, and quinine (QN) were dissolved in 70% ethanol. Lumefantrine (LMF) was dissolved in a mixture of Triton X-100, linoleic acid, and absolute ethanol (in a ratio of 1:1:1). Piperaquine (PQ) was dissolved in 0.5% lactic acid. Drug suspensions were transferred to 96-well flat-bottom plates and air dried under sterile condition.

*P. falciparum* isolates were exposed to CQ (0–2,992.34 nM), MQ (0–370.73 nM), AMQ (0–105.8 nM), QN (0–3,1135.90 nM), and LMF (0–434.83 nM) briefly before the rosetting assay. The experiments were repeated with *P. vivax*, with an additional drug candidate, AS (0–49.42 nM). A set of *P. falciparum* isolates with known K13 single nucleotide polymorphism status and AS-parasite clearance half-life (AS-PCt_1/2_, i.e., time estimated for AS to decrease the patient’s parasitemia by half during the log-linear phase of parasite clearance after administration of AS^4^) was used. Before the rosetting assay, the parasites were briefly exposed to MQ (0–3627.22 nM; higher concentrations than the earlier experiments were used due to the rapid development of MQ resistance among the *P. falciparum* isolates in the area under study^11^^,^^13^) and PQ (0–2469.10 nM). The changes in rosetting rates by AS (49.42 nM) in combination with its partner drugs were evaluated by using the highest concentration point of respective drugs.

Analyses were performed with GraphPad Prism 9.0. Normality of dataset was evaluated using Shapiro-Wilk test. Multiple comparisons of normally distributed data sets were performed using one-way analysis of variance with Dunnett’s multiple comparison test (comparison against drug-free control) and Tukey’s multiple comparison test (cross-group comparisons).

Rosetting rates of *P. falciparum* were significantly increased by CQ, MQ, AMQ, QN, and LMF (Figure 1A–E). A similar trend was found with *P. vivax* (Figure 2A–E). The rosetting rates of *P. vivax* were significantly increased after brief AS exposure (Figure 2F). For the assessment of partner drugs involved in ACT, *P. falciparum* isolates experienced a significant increase in rosetting rates after exposure to MQ (Figure 3A) and PQ (Figure 3B). The concentration range of MQ in Figure 3A was higher than that of Figure 1B. Nevertheless, the findings from both sets of experiments were in agreement. The combination of AS with MQ and PQ did not prevent the rosette-stimulating effect on *P. falciparum* (Figure 3C). However, there were differences in the degrees of rosette-stimulation between the short and long AS-PCt_1/2_ groups. For *P. falciparum* isolates with short AS-PCt_1/2_ (i.e., AS sensitive), no significant difference in the degree of rosette-stimulation was found between the settings with combined drug exposure (AS + MQ and AS + PQ) and those with single drug exposure (AS, MQ, or PQ). For the long AS-PCt_1/2_ (i.e., AS-resistant) group, the degree of rosette stimulation by brief AS exposure was higher than that of MQ but not significantly different from that of PQ. When AS was added with the partner drugs, the rosette-stimulating effect was higher than the conditions with single drug exposure to either of the compounds under study.

**Figure 1.**
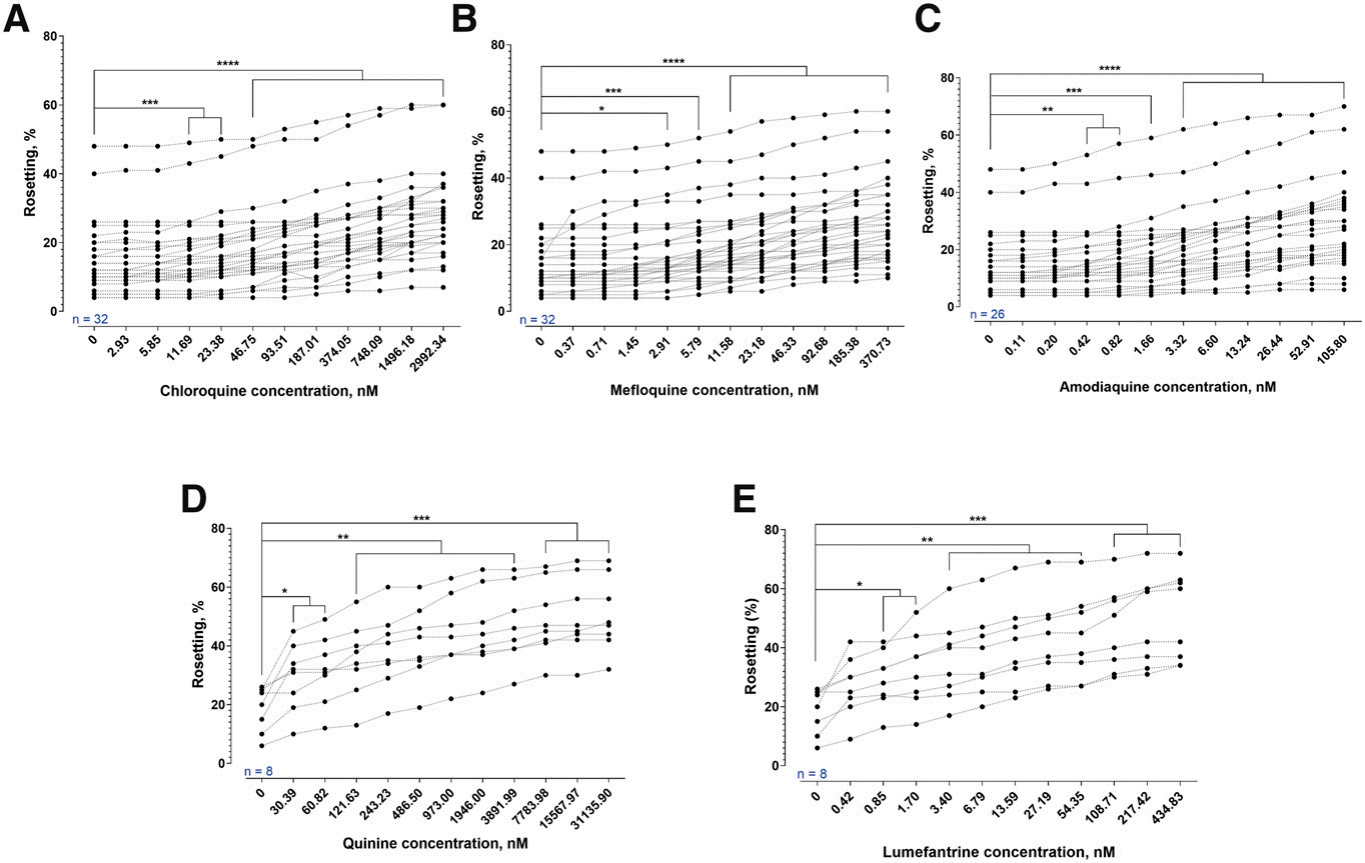
The effects of different antimalarials on rosetting of *P. falciparum*. The sample size for each experiment is shown in blue. One-way analysis of variance with Dunnett’s multiple comparison test was performed to compare the rosetting rates recorded from drug-exposed settings with that of drug-free control. (**A**) Chloroquine significantly increased rosetting rates at ≥ 11.69 nM (*P* = 0.008, 0.0001 for 11.69 nM and 23.38 nM, respectively; *P* < 0.0001 for 46.75–2992.34 nM). (**B**) Mefloquine induced significant increment of rosetting rates at 2.91 nM (*P* = 0.0116), 5.79 nM (*P* = 0.0002), and 11.56 through 370.73 nM (*P* < 0.0001). (**C**) Amodiaquine exerted significant rosette stimulation at 0.42 nM and above (*P* = 0.0049, 0.001, 0.002 for 0.42 nM; 0.82 nM; and 1.66 nM, respectively; *P* < 0.0001 for 3.32–105.8 nM). (**D**) Quinine exerted significant stimulation at 30.39 nM and above (*P* = 0.0456, 0.0204, 0.0095, 0.0044, 0.0029, 0.0025, 0.0028, 0.0014, 0.0007, 0.0006, and 0.0005 for 30.39, 60.82, 121.63, 243.23, 486.50, 973.00, 1946.00, 3891.99, 7783.98, 15567.97, and 31135.90 nM, respectively). (**E**) Lumefantrine (LMF) significantly increased rosetting rates at concentration points of 0.85 nM and above (*P* = 0.0118, 0.0108, 0.0099, 0.0078, 0.0041, 0.0022, 0.0019, 0.0007, 0.0006, 0.0005 for 0.85, 1.70, 3.40, 6.79, 13.59, 27.19, 54.35, 108.71, 217.42, and 434.83 nM, respectively).

**Figure 2.**
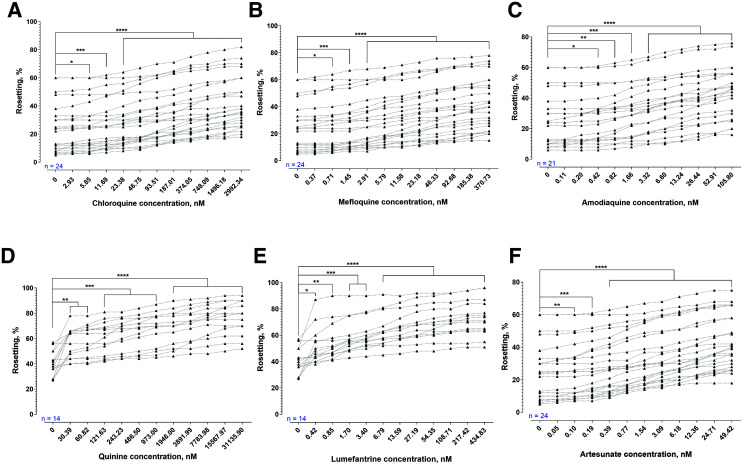
The effects of different antimalarials on rosetting of *P. vivax*. The sample size for each experiment is shown in blue. One-way analysis of variance with Dunnett’s multiple comparison test was performed to compare the rosetting rates recorded from drug-exposed settings with that of drug-free control. (**A**) Chloroquine significantly increased rosetting rates from concentration points of 5.85 nM and above (*P* = 0.0176, 0.0008, 0.0008 for 5.85, 11.69, and 23.38 nM, respectively; *P* < 0.0001 for 46.75–2,992.34 nM). (**B**) Mefloquine significantly increased rosetting rates at ≥ 0.71 nM (*P* = 0.0367 and 0.0008 for 0.71 and 1.45 nM, respectively; *P* < 0.0001 for 2.91–370.73 nM). (**C**) AMQ increased rosetting rates at ≥ 0.42 nM (*P* = 0.0412, 0.0022, 0.0004 for 0.42, 0.82, and 1.66 nM, respectively; *P* < 0.0001 for 3.32–105.80 nM). (**D**) Quinine (QN) exerted significant rosette-stimulation at ≥ 30.39 nM (*P* = 0.0026, 0.0015, 0.0009, 0.0005, 0.0002, 0.0001 for 30.39, 60.82, 121.63, 243.23, 486.50, and 973 nM, respectively; *P* < 0.0001 for 1,946–31,135.90 nM). (**E**) Lumefantrine induced significant changes at ≥ 0.42 nM (*P* = 0.0208, 0.0042, 0.0004, 0.0001 for 0.42, 0.85, 1.70, and 3.40 nM, respectively; *P* < 0.0001 for 6.79–434.83 nM). (**F**) Significant rosetting rate increment was observed with artesunate at ≥ 0.05 nM (*P* = 0.0373, 0.0041, 0.0005 for 0.05, 0.10, and 0.19 nM, respectively; *P* < 0.0001 for 0.39–49.42 nM).

**Figure 3.**
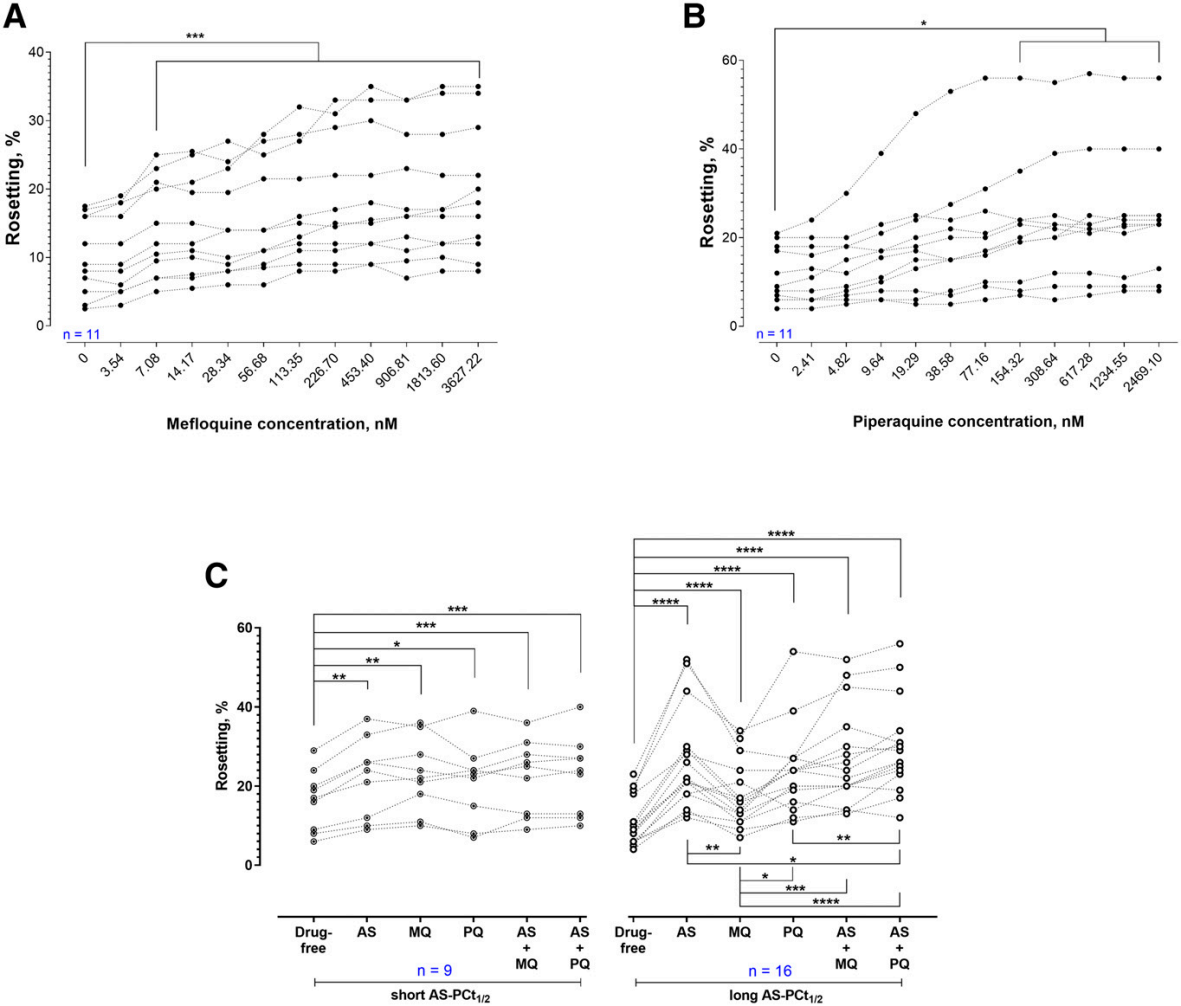
The rosette-stimulatory effect of artemisinin compounds. (**A**) Effect of mefloquine (MQ) on rosetting. Compared with control, a significant difference was found at drug concentration points of ≥ 28.34 nM (one-way analysis of variance [ANOVA] with Dunnett’s test *P* = 0.0231, 0.0071, 0.0074, 0.0035, 0.0024, 0.0011, 0.0009, and 0.0006, respectively). (**B**) Effect of piperaquine (PQ) on rosetting. Compared with control, a significant difference was found at drug concentration points of ≥ 154.32 nM (one-way ANOVA with Dunnett’s test *P* = 0.0318, 0.0197, 0.0185, 0.0136, and 0.0116, respectively). (**C**) Rosetting of isolates after exposure to artesunate (AS), MQ, PQ, AS + MQ, and AS + PQ. One way ANOVA with Tukey’s test was conducted. For the short AS-PCt_1/2_ group, rosetting rates were increased by AS, MQ, PQ, AS + MQ, and AS + PQ (*P* = 0.0019, 0.0014, 0.0257, 0.0002, and 0.0003, respectively). No significant difference found between AS with AS+MQ and AS + PQ (*P* = 0.9056 and 0.7255, respectively). For long AS-PCt_1/2_ group, rosetting rates were increased by AS, MQ, PQ, AS + MQ, and AS + PQ (*P* < 0.0001 for all). *P* = 0.0162 and 0.9761 for AS vs AS + PQ and AS vs. AS + MQ, respectively. No significant difference was found between AS + PQ and AS + MQ (*P* = 0.0762).

The phenomenon of drug-exposure-induced rosetting is not specific to ART and its derivatives. An earlier study suggested that as soon as antimalarial drugs enter the IRBC, they trigger an immediate shock-like signal to the intracellular parasite, which leads to the induction of rapid rosette formation mediated by the parasite’s protein trafficking machinery from the IRBC cytoplasm to the surface of IRBC.^4^ Such a response is probably conserved across species, at least for *P. falciparum* and *P. vivax*, based on our findings. Notably, all drug compounds in this study have good membrane permeability at physiologic pH condition,^14^ which may contribute to the observed rapid rosette-stimulation by the parasites. In fact, most of the antimalarials available on the market have good membrane permeability.^14^ Nevertheless, we do not expect all drugs to stimulate *Plasmodium* spp. rosette formation. Drug compounds with low lipophilicity may not be readily membrane permeable and hence are unlikely to induce rosette-stimulation. Furthermore, drug compounds that can rapidly rigidify IRBC may destroy all cytoadherence properties of IRBC.

The prevalence of rosetting rates varies with geographic origin. For example, the prevalence of rosetting is higher in cerebral malaria samples from some African countries.^15^^,^^16^ Outside Africa, rosetting phenomenon is relatively common for *P. falciparum* and *P. vivax* (both severe and uncomplicated malaria) from Thailand and Papua New Guinea.^17^^–^^19^ Interestingly, the Greater Mekong Subregion (GMS) of Southeast Asia has been the epicenter of treatment-resistant malaria against several antimalarials such as ART and its derivatives CQ and MFQ.^8^ Of note, the rosetting ligand of *P. vivax* has yet to be determined. On the other hand, the expression of *P. falciparum* rosetting ligand PfEMP1 among the Southeast Asian *P. falciparum* isolates was suggested to be associated with the natural selection process of a resistance phenotype against ART and other antimalarial compounds.^20^ Drug-mediated rosette stimulation may be a reflection of this selection process in the parasite population of this geographic area. The ability to rosette more upon drug (threat) encounter may facilitate the parasite population to select genotypes that give rise to more specific and efficient strategies against a particular drug. The “priming” by different drugs drives better and faster adaptation of these parasites to new treatment regimens introduced to this area.

Drug-mediated rosetting is a relatively common feature in *P. falciparum* and *P. vivax* isolates from Thailand. The reflex-like response by the parasites upon drug exposure may help them to survive a brief encounter with a harmful environment. More studies are needed to evaluate the potential of a drug-mediated rosetting assay as an economic method to monitor or predict drug resistance development in the parasite population.

## Supplemental Material


Supplemental materials

## References

[1] Lee WC Russell B Rénia L , 2019. Sticking for a cause: the falciparum malaria parasites cytoadherence paradigm. Front Immunol 10: 1444. 31316507 10.3389/fimmu.2019.01444 PMC6610498

[2] Lee WC 2020. *Plasmodium*-infected erythrocytes induce secretion of IGFBP7 to form type II rosettes and escape phagocytosis. eLife 9: e51546. 32066522 10.7554/eLife.51546 PMC7048393

[3] Albrecht L 2020. Rosettes integrity protects *Plasmodium vivax* of being phagocytized. Sci Rep 10: 16706. 33028898 10.1038/s41598-020-73713-w PMC7541459

[4] Lee WC Russell B Lee B Chu CS Phyo AP Sriprawat K Lau YL Nosten F Rénia L , 2021. *Plasmodium falciparum* rosetting protects schizonts against artemisinin. EBioMedicine 73: 103680. 34749300 10.1016/j.ebiom.2021.103680 PMC8586750

[5] Borlon C Russell B Sriprawat K Suwanarusk R Erhart A Renia L Nosten F D’Alessandro U , 2012. Cryopreserved *Plasmodium vivax* and cord blood reticulocytes can be used for invasion and short term culture. Int J Parasitol 42: 155–160. 22240310 10.1016/j.ijpara.2011.10.011 PMC3438882

[6] Lee WC Russell B Lau YL Fong MY Chu C Sriprawat K Suwanarusk R Nosten F Renia L , 2013. Giemsa-stained wet mount based method for reticulocyte quantification: a viable alternative in resource limited or malaria endemic settings. PLoS One 8: e60303. 23565221 10.1371/journal.pone.0060303 PMC3614967

[7] Lee WC Rénia L , 2020. Microscopy-based methods for rosetting assay in malaria research. Bio Protoc 10: e3665. 10.21769/BioProtoc.3665 PMC7842651 33659335

[8] Phompradit P Muhamad P Wisedpanichkij R Chaijaroenkul W Na-Bangchang K , 2014. Four years’ monitoring of in vitro sensitivity and candidate molecular markers of resistance of *Plasmodium falciparum* to artesunate-mefloquine combination in the Thai–Myanmar border. Malar J 13: 23. 24423390 10.1186/1475-2875-13-23 PMC3896708

[9] Childs GE Pang L Wimonwattrawatee T Pooyindee N Nanakorn A Limchitee S Webster HK , 1987. In vitro mefloquine resistance of *Plasmodium falciparum* isolated from the burmese border region of Thailand. Southeast Asian J Trop Med Public Health 18: 438–443. 3329409

[10] Childs GE Häusler B Milhous W Chen C Wimonwattrawatee T Pooyindee N Boudreau EF , 1988. In vitro activity of pyronaridine against field isolates and reference clones of *Plasmodium falciparum.* Am J Trop Med Hyg 38: 24–29. 3277460 10.4269/ajtmh.1988.38.24

[11] Barends M Jaidee A Khaohirun N Singhasivanon P Nosten F , 2007. *In vitro* activity of ferroquine (ssr 97193) against *Plasmodium falciparum* isolates from the Thai–Burmese border. Malar J 6: 81. 17597537 10.1186/1475-2875-6-81 PMC1934364

[12] Carrara VI 2006. Deployment of early diagnosis and mefloquine-artesunate treatment of falciparum malaria in Thailand: the tak malaria initiative. PLoS Med 3: e183. 16719547 10.1371/journal.pmed.0030183 PMC1470664

[13] Rojanawatsirivet C Congpuong K Vijaykadga S Thongphua S Thongsri K Bangchang KN Wilairatana P Wernsdorfer WH , 2004. Declining mefloquine sensitivity of *Plasmodium falciparum* along the Thai–Myanmar border. Southeast Asian J Trop Med Public Health 35: 560–565. 15689066

[14] Basore K Cheng Y Kushwaha AK Nguyen ST Desai SA , 2015. How do antimalarial drugs reach their intracellular targets? Front Pharmacol 6: 91. 25999857 10.3389/fphar.2015.00091 PMC4419668

[15] Doumbo OK Thera MA Koné AK Raza A Tempest LJ Lyke KE Plowe CV Rowe JA , 2009. High levels of *Plasmodium falciparum* rosetting in all clinical forms of severe malaria in African children. Am J Trop Med Hyg 81: 987–993. 19996426 10.4269/ajtmh.2009.09-0406 PMC2877664

[16] Rogerson SJ Beeson JG Mhango CG Dzinjalamala FK Molyneux ME , 2000. *Plasmodium falciparum* rosette formation is uncommon in isolates from pregnant women. Infect Immun 68: 391–393. 10603414 10.1128/iai.68.1.391-393.2000 PMC97147

[17] al-Yaman F Genton B Mokela D Raiko A Kati S Rogerson S Reeder J Alpers M , 1995. Human cerebral malaria: lack of significant association between erythrocyte rosetting and disease severity. Trans R Soc Trop Med Hyg 89: 55–58. 7747308 10.1016/0035-9203(95)90658-4

[18] Lee WC 2014. Glycophorin C (CD236R) mediates vivax malaria parasite rosetting to normocytes. Blood 123: e100–e109. 24652986 10.1182/blood-2013-12-541698 PMC4007619

[19] Ho M Davis TM Silamut K Bunnag D White NJ , 1991. Rosette formation of *Plasmodium falciparum*–infected erythrocytes from patients with acute malaria. Infect Immun 59: 2135–2139. 2037374 10.1128/iai.59.6.2135-2139.1991 PMC257977

[20] Otto TD Assefa SA Böhme U Sanders MJ Kwiatkowski D Berriman M Newbold C , 2019. Evolutionary analysis of the most polymorphic gene family in falciparum malaria. Wellcome Open Res 4: 193. 32055709 10.12688/wellcomeopenres.15590.1 PMC7001760

