# Supplementary material for: Rosetting Responses of Plasmodium-infected Erythrocytes to Antimalarials
Source: Am J Trop Med Hyg. 2022 Apr 11;106(6):1670–4. doi: 10.4269/ajtmh.21-1229 (PMC9209907; doi:10.4269/ajtmh.21-1229)
Supplement: Supplementary file 1 [file tpmd211229.SD1.pdf]

Supplemental Table 1. Reagents and tools table

Reagent type (species) or resource	Designation	Source or reference	Identifiers	Additional information
Biological sample (*Plasmodium falciparum*)	PID412725	Lee *et al.*, 2021. doi: 10.1016/j.ebiom.2021.103680		Fresh clinical isolate from the North western part of Thailand<br><br>Date of collection: Apr 2013<br><br>Used in experiments for figures 1A, 1B, 1C
Biological sample (*Plasmodium falciparum*)	PID405104	Lee *et al.*, 2021. doi: 10.1016/j.ebiom.2021.103680		Fresh clinical isolate from the North western part of Thailand<br><br>Date of collection: Apr 2013<br><br>Used in experiments for figures 1A, 1B, 1C
Biological sample (*Plasmodium falciparum*)	PID403525	Lee *et al.*, 2021. doi: 10.1016/j.ebiom.2021.103680		Fresh clinical isolate from the North western part of Thailand<br><br>Date of collection: Apr 2013<br><br>Used in experiments for figures 1A, 1B, 1C
Biological sample (*Plasmodium falciparum*)	PID109053	Lee *et al.*, 2021. doi: 10.1016/j.ebiom.2021.103680		Fresh clinical isolate from the North western part of Thailand<br><br>Date of collection: Apr 2013<br><br>Used in experiments for figures 1A, 1B, 1C
Biological sample (*Plasmodium falciparum*)	PID102938	Lee *et al.*, 2021. doi: 10.1016/j.ebiom.2021.103680		Fresh clinical isolate from the North western part of Thailand<br><br>Date of collection: Apr 2013<br><br>Used in experiments for figures 1A, 1B, 1C

Biological sample (*Plasmodium falciparum*)	PID402420	Lee *et al.*, 2021. doi: 10.1016/j.ebiom.2021.103680		Fresh clinical isolate from the North western part of Thailand<br><br>Date of collection: Apr 2013<br><br>Used in experiments for figures 1A, 1B, 1C
Biological sample (*Plasmodium falciparum*)	PID402635	Lee *et al.*, 2021. doi: 10.1016/j.ebiom.2021.103680		Fresh clinical isolate from the North western part of Thailand<br><br>Date of collection: Apr 2013<br><br>Used in experiments for figures 1A, 1B, 1C
Biological sample (*Plasmodium falciparum*)	PID412788	Lee *et al.*, 2021. doi: 10.1016/j.ebiom.2021.103680		Fresh clinical isolate from the North western part of Thailand<br><br>Date of collection: Apr 2013<br><br>Used in experiments for figures 1A, 1B, 1C
Biological sample (*Plasmodium falciparum*)	PID305649	Lee *et al.*, 2021. doi: 10.1016/j.ebiom.2021.103680		Fresh clinical isolate from the North western part of Thailand<br><br>Date of collection: Apr 2013<br><br>Used in experiments for figures 1A, 1B, 1C
Biological sample (*Plasmodium falciparum*)	PID412890	Lee *et al.*, 2021. doi: 10.1016/j.ebiom.2021.103680		Fresh clinical isolate from the North western part of Thailand<br><br>Date of collection: Apr 2013<br><br>Used in experiments for figures 1A, 1B, 1C
Biological sample (*Plasmodium falciparum*)	PID404033	Lee *et al.*, 2021. doi: 10.1016/j.ebiom.2021.103680		Fresh clinical isolate from the North western part of Thailand

				Date of collection: May 2013<br><br>Used in experiments for figures 1A, 1B, 1C
Biological sample (*Plasmodium falciparum*)	PID412895	Lee *et al*., 2021. doi: 10.1016/j.ebiom.2021.103680		Fresh clinical isolate from the North western part of Thailand<br><br>Date of collection: May 2013<br><br>Used in experiments for figures 1A, 1B, 1C
Biological sample (*Plasmodium falciparum*)	PID412920	Lee *et al*., 2021. doi: 10.1016/j.ebiom.2021.103680		Fresh clinical isolate from the North western part of Thailand<br><br>Date of collection: May 2013<br><br>Used in experiments for figures 1A, 1B, 1C
Biological sample (*Plasmodium falciparum*)	PID404184	Lee *et al*., 2021. doi: 10.1016/j.ebiom.2021.103680		Fresh clinical isolate from the North western part of Thailand<br><br>Date of collection: May 2013<br><br>Used in experiments for figures 1A, 1B, 1C
Biological sample (*Plasmodium falciparum*)	PID412962	Lee *et al*., 2021. doi: 10.1016/j.ebiom.2021.103680		Fresh clinical isolate from the North western part of Thailand<br><br>Date of collection: May 2013<br><br>Used in experiments for figures 1A, 1B, 1C
Biological sample (*Plasmodium falciparum*)	PID412977	Lee *et al*., 2021. doi: 10.1016/j.ebiom.2021.103680		Fresh clinical isolate from the North western part of Thailand<br><br>Date of collection: May 2013<br><br>Used in experiments for figures 1A, 1B,

				1C
Biological sample (*Plasmodium falciparum*)	PID401322	Lee *et al.*, 2021. doi: 10.1016/j.ebiom.2021.103680		Fresh clinical isolate from the North western part of Thailand<br><br>Date of collection: May 2013<br><br>Used in experiments for figures 1A, 1B, 1C
Biological sample (*Plasmodium falciparum*)	PID409347	Lee *et al.*, 2021. doi: 10.1016/j.ebiom.2021.103680		Fresh clinical isolate from the North western part of Thailand<br><br>Date of collection: May 2013<br><br>Used in experiments for figures 1A, 1B, 1C
Biological sample (*Plasmodium falciparum*)	PID411792	Lee *et al.*, 2021. doi: 10.1016/j.ebiom.2021.103680		Fresh clinical isolate from the North western part of Thailand<br><br>Date of collection: May 2013<br><br>Used in experiments for figures 1A, 1B, 1C
Biological sample (*Plasmodium falciparum*)	PID402127	Lee *et al.*, 2021. doi: 10.1016/j.ebiom.2021.103680		Fresh clinical isolate from the North western part of Thailand<br><br>Date of collection: May 2013<br><br>Used in experiments for figures 1A, 1B, 1C
Biological sample (*Plasmodium falciparum*)	PID413003	Lee *et al.*, 2021. doi: 10.1016/j.ebiom.2021.103680		Fresh clinical isolate from the North western part of Thailand<br><br>Date of collection: May 2013<br><br>Used in experiments for figures 1A, 1B, 1C
Biological sample (*Plasmodium falciparum*)	PID109190	Lee *et al.*, 2021. doi: 10.1016/j.ebiom.2021.103680		Fresh clinical isolate from the North western part of Thailand

				Date of collection: May 2013<br><br>Used in experiments for figures 1A, 1B, 1C
Biological sample (*Plasmodium falciparum*)	MMA30037	Lee *et al*., 2021. doi: 10.1016/j.ebiom.2021.103680		Fresh clinical isolate from the North western part of Thailand<br><br>Date of collection: May 2013<br><br>Used in experiments for figures 1A, 1B, 1C
Biological sample (*Plasmodium falciparum*)	NHP3219	Lee *et al*., 2021. doi: 10.1016/j.ebiom.2021.103680		Fresh clinical isolate from the North western part of Thailand<br><br>Date of collection: May 2013<br><br>Used in experiments for figures 1A, 1B, 1C
Biological sample (*Plasmodium falciparum*)	MMA30038	Lee *et al*., 2021. doi: 10.1016/j.ebiom.2021.103680		Fresh clinical isolate from the North western part of Thailand<br><br>Date of collection: May 2013<br><br>Used in experiments for figures 1A, 1B, 1C
Biological sample (*Plasmodium falciparum*)	PID413024	Lee *et al*., 2021. doi: 10.1016/j.ebiom.2021.103680		Fresh clinical isolate from the North western part of Thailand<br><br>Date of collection: May 2013<br><br>Used in experiments for figures 1A, 1B, 1C
Biological sample (*Plasmodium falciparum*)	MMA40095	Lee *et al*., 2021. doi: 10.1016/j.ebiom.2021.103680		Fresh clinical isolate from the North western part of Thailand<br><br>Date of collection: May 2013<br><br>Used in experiments

				for figures 1A, 1B
Biological sample (*Plasmodium falciparum*)	PID413073	Lee *et al.*, 2021. doi: 10.1016/j.ebiom.2021.103680		Fresh clinical isolate from the North western part of Thailand<br><br>Date of collection: May 2013<br><br>Used in experiments for figures 1A, 1B
Biological sample (*Plasmodium falciparum*)	PID412793	Lee *et al.*, 2021. doi: 10.1016/j.ebiom.2021.103680		Fresh clinical isolate from the North western part of Thailand<br><br>Date of collection: May 2013<br><br>Used in experiments for figures 1A, 1B
Biological sample (*Plasmodium falciparum*)	PID109262	Lee *et al.*, 2021. doi: 10.1016/j.ebiom.2021.103680		Fresh clinical isolate from the North western part of Thailand<br><br>Date of collection: May 2013<br><br>Used in experiments for figures 1A, 1B
Biological sample (*Plasmodium falciparum*)	MMA30040	Lee *et al.*, 2021. doi: 10.1016/j.ebiom.2021.103680		Fresh clinical isolate from the North western part of Thailand<br><br>Date of collection: May 2013<br><br>Used in experiments for figures 1A, 1B
Biological sample (*Plasmodium falciparum*)	PID413071	Lee *et al.*, 2021. doi: 10.1016/j.ebiom.2021.103680		Fresh clinical isolate from the North western part of Thailand<br><br>Date of collection: May 2013<br><br>Used in experiments for figures 1A, 1B
Biological sample (*Plasmodium falciparum*)	PID403035	This paper		Fresh clinical isolate from the North western part of Thailand<br><br>Date of collection: May 2013<br><br>Used in experiments

				for figures 1D, 1E
Biological sample (*Plasmodium falciparum*)	PID408028	This paper		Fresh clinical isolate from the North western part of Thailand<br><br>Date of collection: May 2013<br><br>Used in experiments for figures 1D, 1E
Biological sample (*Plasmodium falciparum*)	RDM00036	This paper		Fresh clinical isolate from the North western part of Thailand<br><br>Date of collection: May 2013<br><br>Used in experiments for figures 1D, 1E
Biological sample (*Plasmodium falciparum*)	MMA3050	This paper		Fresh clinical isolate from the North western part of Thailand<br><br>Date of collection: May 2013<br><br>Used in experiments for figures 1D, 1E
Biological sample (*Plasmodium falciparum*)	MMA2156	This paper		Fresh clinical isolate from the North western part of Thailand<br><br>Date of collection: May 2013<br><br>Used in experiments for figures 1D, 1E
Biological sample (*Plasmodium falciparum*)	MMA4044	This paper		Fresh clinical isolate from the North western part of Thailand<br><br>Date of collection: May 2013<br><br>Used in experiments for figures 1D, 1E
Biological sample (*Plasmodium falciparum*)	MMA2104	This paper		Fresh clinical isolate from the North western part of Thailand<br><br>Date of collection: May 2013<br><br>Used in experiments

				for figures 1D, 1E
Biological sample (*Plasmodium falciparum*)	RDM00037	This paper		Fresh clinical isolate from the North western part of Thailand<br><br>Date of collection: May 2013<br><br>Used in experiments for figures 1D, 1E
Biological sample (*Plasmodium falciparum*)	NHP1450	Lee *et al*., 2021. doi: 10.1016/j.ebiom.2021.103680		Clinical isolate from the North western part of Thailand<br>AS-PC$t_{1/2}$ = 8.87 hr; K13_N458Y<br><br>Date of collection: Jun 2012<br><br>Used in experiments for figures 3A, 3B, 3C
Biological sample (*Plasmodium falciparum*)	NHP4770	Lee *et al*.2020. doi: 10.7554/3Life.51546		Adapted from clinical isolate in SMRU<br>AS-PC$t_{1/2}$ = 8.07 hr; K13_A675V<br><br>Date of collection: Feb 2012<br><br>Used in experiments for figures 3A, 3B, 3C
Biological sample (*Plasmodium falciparum*)	NHP2203	Lee *et al*., 2021. doi: 10.1016/j.ebiom.2021.103680		Clinical isolate from the North western part of Thailand<br>AS-PC$t_{1/2}$ = 8.83 hr; K13_R575K<br><br>Date of collection: Jun 2013<br><br>Used in experiments for figures 3A, 3B, 3C
Biological sample (*Plasmodium falciparum*)	NHP1403	Lee *et al*., 2021. doi: 10.1016/j.ebiom.2021.103680		Clinical isolate from the North western part of Thailand<br>AS-PC$t_{1/2}$ = 7.7 hr; K13_A675V<br><br>Date of collection: Feb 2012<br><br>Used in

				experiments for figures 3A, 3B, 3C
Biological sample (*Plasmodium falciparum*)	NHP1481	Lee *et al.*, 2021. doi: 10.1016/j.ebiom.2021.103680		Clinical isolate from the North western part of Thailand<br>AS-PCt$_{1/2}$ = 7.96 hr; K13_R561H<br><br>Date of collection: Jul 2012<br><br>Used in experiments for figures 3A, 3B, 3C
Biological sample (*Plasmodium falciparum*)	NHP4939	Lee *et al.*, 2021. doi: 10.1016/j.ebiom.2021.103680		Clinical isolate from the North western part of Thailand<br>AS-PCt$_{1/2}$ = 8.77 hr; K13_C580Y<br><br>Date of collection: Sep 2013<br><br>Used in experiments for figures 3A, 3B, 3C
Biological sample (*Plasmodium falciparum*)	NHP4722	Lee *et al.*, 2021. doi: 10.1016/j.ebiom.2021.103680		Clinical isolate from the North western part of Thailand<br>AS-PCt$_{1/2}$ = 8.6 hr; K13_A675V<br><br>Date of collection: May 2011<br><br>Used in experiments for figures 3A, 3B, 3C
Biological sample (*Plasmodium falciparum*)	OHP646	Lee *et al.*, 2021. doi: 10.1016/j.ebiom.2021.103680		Clinical isolate from the North western part of Thailand<br>AS-PCt$_{1/2}$ = 1.58 hr; K13_WT<br><br>Date of collection: Jul 2002<br><br>Used in experiments for figures 3A, 3B, 3C
Biological sample (*Plasmodium falciparum*)	NHP3048	Lee *et al.*, 2021. doi: 10.1016/j.ebiom.2021.103680		Clinical isolate from the North western part of Thailand<br>AS-PCt$_{1/2}$ = 1.91 hr; K13_WT

				Date of collection: Aug 2008<br><br>Used in experiments for figures 3A, 3B, 3C
Biological sample (*Plasmodium falciparum*)	OHP319	Lee *et al*., 2021. doi: 10.1016/j.ebiom.2021.103680		Clinical isolate from the North western part of Thailand<br>AS-PC$t_{1/2}$ = 1.9 hr; K13_WT<br><br>Date of collection: Jun 2004<br><br>Used in experiments for figures 3A, 3B, 3C
Biological sample (*Plasmodium falciparum*)	NHP1106	Lee *et al*.2020. doi: 10.7554/3Life.51546		Adapted from clinical isolate in SMRU<br>AS-PC$t_{1/2}$ = 1.71 hr; K13_WT<br><br>Date of collection: Oct 2008<br><br>Used in experiments for figures 3A, 3B, 3C
Biological sample (*Plasmodium falciparum*)	NHP2037	Lee *et al*., 2021. doi: 10.1016/j.ebiom.2021.103680		Clinical isolate from the North western part of Thailand<br>AS-PC$t_{1/2}$ = 1.82 hr; K13_WT<br><br>Date of collection: May 2008<br><br>Used in experiments for figure 3C
Biological sample (*Plasmodium falciparum*)	OHP602	Lee *et al*., 2021. doi: 10.1016/j.ebiom.2021.103680		Clinical isolate from the North western part of Thailand<br>AS-PC$t_{1/2}$ = 1.04 hr; K13_WT<br><br>Date of collection: Mar 2002<br><br>Used in experiments for figure 3C
Biological sample	NHP4703	Lee *et al*., 2021. doi: 10.1016/j.ebiom.2021.103		Clinical isolate from the North

(*Plasmodium falciparum*)		680		western part of Thailand<br>AS-PC$t_{1/2}$ = 1.2 hr; K13_WT<br><br>Date of collection: Apr 2011<br><br>Used in experiments for figure 3C
Biological sample (*Plasmodium falciparum*)	NHP1386	Lee *et al.*, 2021. doi: 10.1016/j.ebiom.2021.103680		Clinical isolate from the North western part of Thailand<br>AS-PC$t_{1/2}$ = 1.67 hr; K13_WT<br><br>Date of collection: Jul 2011<br><br>Used in experiments for figure 3C
Biological sample (*Plasmodium falciparum*)	OHP590	Lee *et al.*, 2021. doi: 10.1016/j.ebiom.2021.103680		Clinical isolate from the North western part of Thailand<br><br>Date of collection: Jan 2002<br><br>AS-PC$t_{1/2}$ = 1.23 hr; K13_WT<br><br>Used in experiments for figure 3C
Biological sample (*Plasmodium falciparum*)	NHP1401	Lee *et al.*, 2021. doi: 10.1016/j.ebiom.2021.103680		Clinical isolate from the North western part of Thailand<br>AS-PC$t_{1/2}$ = 9.18 hr; K13_C580Y<br><br>Date of collection: Feb 2012<br><br>Used in experiments for figure 3C
Biological sample (*Plasmodium falciparum*)	NHP1507	Lee *et al.*, 2021. doi: 10.1016/j.ebiom.2021.103680		Clinical isolate from the North western part of Thailand<br>AS-PC$t_{1/2}$ = 9.24 hr; K13_R561H<br><br>Date of collection: Jun 2013

				Used in experiments for figure 3C
Biological sample (*Plasmodium falciparum*)	NHP1461	Lee *et al.*, 2021. doi: 10.1016/j.ebiom.2021.103680		Clinical isolate from the North western part of Thailand<br>AS-PCt$_{1/2}$ = 9.21 hr; K13_P441L<br><br>Date of collection: Jun 2012<br><br>Used in experiments for figure 3C
Biological sample (*Plasmodium falciparum*)	NHP3127	Lee *et al.*, 2021. doi: 10.1016/j.ebiom.2021.103680		Clinical isolate from the North western part of Thailand<br>AS-PCt$_{1/2}$ = 9.11 hr; K13_R561H<br><br>Date of collection: Jun 2010<br><br>Used in experiments for figure 3C
Biological sample (*Plasmodium falciparum*)	NHP1454	Lee *et al.*, 2021. doi: 10.1016/j.ebiom.2021.103680		Clinical isolate from the North western part of Thailand<br>AS-PCt$_{1/2}$ = 7.98 hr; K13_R561H<br><br>Date of collection: Jun 2012<br><br>Used in experiments for figure 3C
Biological sample (*Plasmodium falciparum*)	NHP3160	Lee *et al.*, 2021. doi: 10.1016/j.ebiom.2021.103680		Clinical isolate from the North western part of Thailand<br>AS-PCt$_{1/2}$ = 8.59 hr; K13_N458Y<br><br>Date of collection: Jun 2011<br><br>Used in experiments for figure 3C
Biological sample (*Plasmodium falciparum*)	NHP1455	Lee *et al.*, 2021. doi: 10.1016/j.ebiom.2021.103680		Clinical isolate from the North western part of Thailand

				AS-PCt$_{1/2}$ = 8.02 hr; K13_R561H<br><br>Date of collection: Jun 2012<br><br>Used in experiments for figure 3C
Biological sample (*Plasmodium falciparum*)	NHP1332	Lee *et al*., 2021. doi: 10.1016/j.ebiom.2021.103680		Clinical isolate from the North western part of Thailand<br>AS-PCt$_{1/2}$ = 8.54 hr; K13_N458Y<br><br>Date of collection: Apr 2011<br><br>Used in experiments for figure 3C
Biological sample (*Plasmodium falciparum*)	NHP4870	Lee *et al*., 2021. doi: 10.1016/j.ebiom.2021.103680		Clinical isolate from the North western part of Thailand<br>AS-PCt$_{1/2}$ = 8.9 hr; K13_C580Y<br><br>Date of collection: Mar 2013<br><br>Used in experiments for figure 3C
Biological sample (*Plasmodium vivax*)	BPD289	This paper		Fresh clinical isolate from the North western part of Thailand<br><br>Date of collection: Apr 2013<br><br>Used in experiments for figures 2A, 2B, 2C, 2F
Biological sample (*Plasmodium vivax*)	DMA300	This paper		Fresh clinical isolate from the North western part of Thailand<br><br>Date of collection: Apr 2013<br><br>Used in experiments for figures 2A, 2B, 2F
Biological sample (*Plasmodium*	PID305596	This paper		Fresh clinical isolate from the North western part

vivax)				of Thailand<br><br>Date of collection: Apr 2013<br><br>Used in experiments for figures 2A, 2B, 2F
Biological sample (*Plasmodium vivax*)	BPD290	This paper		Fresh clinical isolate from the North western part of Thailand<br><br>Date of collection: Apr 2013<br><br>Used in experiments for figures 2A, 2B, 2F
Biological sample (*Plasmodium vivax*)	PID109105	This paper		Fresh clinical isolate from the North western part of Thailand<br><br>Date of collection: Apr 2013<br><br>Used in experiments for figures 2A, 2B, 2C, 2F
Biological sample (*Plasmodium vivax*)	PID412834	This paper		Fresh clinical isolate from the North western part of Thailand<br><br>Date of collection: Apr 2013<br><br>Used in experiments for figures 2A, 2B, 2C, 2F
Biological sample (*Plasmodium vivax*)	ML0157	This paper		Fresh clinical isolate from the North western part of Thailand<br><br>Date of collection: Apr 2013<br><br>Used in experiments for figures 2A, 2B, 2C, 2F
Biological sample (*Plasmodium vivax*)	PID412846	This paper		Fresh clinical isolate from the North western part of Thailand<br><br>Date of collection: May 2013

				Used in experiments for figures 2A, 2B, 2C, 2F
Biological sample (*Plasmodium vivax*)	PID412931	This paper		Fresh clinical isolate from the North western part of Thailand<br><br>Date of collection: May 2013<br><br>Used in experiments for figures 2A, 2B, 2C, 2F
Biological sample (*Plasmodium vivax*)	PID402054	This paper		Fresh clinical isolate from the North western part of Thailand<br><br>Date of collection: May 2013<br><br>Used in experiments for figures 2A, 2B, 2C, 2F
Biological sample (*Plasmodium vivax*)	PID412339	This paper		Fresh clinical isolate from the North western part of Thailand<br><br>Date of collection: May 2013<br><br>Used in experiments for figures 2A, 2B, 2C, 2F
Biological sample (*Plasmodium vivax*)	PID413076	This paper		Fresh clinical isolate from the North western part of Thailand<br><br>Date of collection: May 2013<br><br>Used in experiments for figures 2A, 2B, 2C, 2F
Biological sample (*Plasmodium vivax*)	PID401466	This paper		Fresh clinical isolate from the North western part of Thailand<br><br>Date of collection: May 2013<br><br>Used in experiments for figures 2A, 2B, 2C, 2F
Biological sample	PID105065	This paper		Fresh clinical isolate from the

(*Plasmodium vivax*)				North western part of Thailand<br><br>Date of collection: May 2013<br><br>Used in experiments for figures 2A, 2B, 2C, 2F
Biological sample (*Plasmodium vivax*)	PID413041	This paper		Fresh clinical isolate from the North western part of Thailand<br><br>Date of collection: May 2013<br><br>Used in experiments for figures 2A, 2B, 2C, 2F
Biological sample (*Plasmodium vivax*)	BPD300	This paper		Fresh clinical isolate from the North western part of Thailand<br><br>Date of collection: May 2013<br><br>Used in experiments for figures 2A, 2B, 2C, 2F
Biological sample (*Plasmodium vivax*)	PID413066	This paper		Fresh clinical isolate from the North western part of Thailand<br><br>Date of collection: May 2013<br><br>Used in experiments for figures 2A, 2B, 2C, 2F
Biological sample (*Plasmodium vivax*)	PID413056	This paper		Fresh clinical isolate from the North western part of Thailand<br><br>Date of collection: May 2013<br><br>Used in experiments for figures 2A, 2B, 2C, 2F
Biological sample (*Plasmodium vivax*)	PID413017	This paper		Fresh clinical isolate from the North western part of Thailand<br><br>Date of collection: May 2013

				Used in experiments for figures 2A, 2B, 2C, 2F
Biological sample (*Plasmodium vivax*)	BPD303	This paper		Fresh clinical isolate from the North western part of Thailand<br><br>Date of collection: May 2013<br><br>Used in experiments for figures 2A, 2B, 2C, 2F
Biological sample (*Plasmodium vivax*)	TK0768	This paper		Fresh clinical isolate from the North western part of Thailand<br><br>Date of collection: May 2013<br><br>Used in experiments for figures 2A, 2B, 2C, 2F
Biological sample (*Plasmodium vivax*)	MR163	This paper		Fresh clinical isolate from the North western part of Thailand<br><br>Date of collection: May 2013<br><br>Used in experiments for figures 2A, 2B, 2C, 2F
Biological sample (*Plasmodium vivax*)	PID406316	This paper		Fresh clinical isolate from the North western part of Thailand<br><br>Date of collection: May 2013<br><br>Used in experiments for figures 2A, 2B, 2C, 2F
Biological sample (*Plasmodium vivax*)	PID413137	This paper		Fresh clinical isolate from the North western part of Thailand<br><br>Date of collection: May 2013<br><br>Used in experiments for figures 2A, 2B, 2C, 2F
Biological	BPD0483	This paper		Fresh clinical

sample (*Plasmodium vivax*)				isolate from the North western part of Thailand<br><br>Date of collection: May 2013<br><br>Used in experiments for figures 2D, 2E
Biological sample (*Plasmodium vivax*)	PID411161	This paper		Fresh clinical isolate from the North western part of Thailand<br><br>Date of collection: May 2013<br><br>Used in experiments for figures 2D, 2E
Biological sample (*Plasmodium vivax*)	PID406704	This paper		Fresh clinical isolate from the North western part of Thailand<br><br>Date of collection: May 2013<br><br>Used in experiments for figures 2D, 2E
Biological sample (*Plasmodium vivax*)	PID112410	This paper		Fresh clinical isolate from the North western part of Thailand<br><br>Date of collection: May 2013<br><br>Used in experiments for figures 2D, 2E
Biological sample (*Plasmodium vivax*)	PID416460	This paper		Fresh clinical isolate from the North western part of Thailand<br><br>Date of collection: May 2013<br><br>Used in experiments for figures 2D, 2E
Biological sample (*Plasmodium vivax*)	PID403915	This paper		Fresh clinical isolate from the North western part of Thailand<br><br>Date of collection: May 2013<br><br>Used in experiments for figures 2D, 2E
Biological	PID112038	This paper		Fresh clinical

sample (*Plasmodium vivax*)				isolate from the North western part of Thailand<br><br>Date of collection: May 2013<br><br>Used in experiments for figures 2D, 2E
Biological sample (*Plasmodium vivax*)	PID416510	This paper		Fresh clinical isolate from the North western part of Thailand<br><br>Date of collection: May 2013<br><br>Used in experiments for figures 2D, 2E
Biological sample (*Plasmodium vivax*)	BPD491	This paper		Fresh clinical isolate from the North western part of Thailand<br><br>Date of collection: May 2013<br><br>Used in experiments for figures 2D, 2E
Biological sample (*Plasmodium vivax*)	BPD493	This paper		Fresh clinical isolate from the North western part of Thailand<br><br>Date of collection: May 2013<br><br>Used in experiments for figures 2D, 2E
Biological sample (*Plasmodium vivax*)	PID403130	This paper		Fresh clinical isolate from the North western part of Thailand<br><br>Date of collection: May 2013<br><br>Used in experiments for figures 2D, 2E
Biological sample (*Plasmodium vivax*)	PID416546	This paper		Fresh clinical isolate from the North western part of Thailand<br><br>Date of collection: May 2013<br><br>Used in experiments for figures 2D, 2E
Biological	BPD494	This paper		Fresh clinical

sample (*Plasmodium vivax*)				isolate from the North western part of Thailand<br><br>Date of collection: May 2013<br><br>Used in experiments for figures 2D, 2E
Biological sample (*Plasmodium vivax*)	PID100197	This paper		Fresh clinical isolate from the North western part of Thailand<br><br>Date of collection: May 2013<br><br>Used in experiments for figures 2D, 2E
Chemical compound, drug	Amodiaquine	Sigma-Aldrich®	Cat # 1031004	
Antibody	Anti-A (ABO1) murine monoclonal antibody	TransClone® Bio-Rad	Cat # 86328	Blood group-typing (slide method: 50 µl)
Antibody	Anti-B (ABO2) murine monoclonal antibody	TransClone® Bio-Rad	Cat # 86470	Blood group-typing (slide method: 50 µl)
Chemical compound, drug	Artesunate	Sigma-Aldrich®	Cat # A3731	
Other	BD Vacutainer™ with lithium heparin	ThermoFisher Scientific	Cat # 02-657-28	
Other	Centrifuge	Sorvall®	Legend® RT Plus	
Other	CF11 cellulose powder	Sigma-Aldrich®	Cat # 318094	
Chemical compound, drug	Chloroquine	Sigma-Aldrich®	Cat # C6628	
Other	Dual CCD digital camera for microscope	Olympus®	Model DP80	
Chemical compound, drug	Ethanol	Sigma-Aldrich®	Cat # E7023	
Other	Falcon® Cell Culture Flask T25, filter cap	VWR™	Cat #29185298	
Other	Flat bottom plate, 96 well	NUNC™	Cat #44240421	
Other	Giemsa	Merck	Cat # HX60416604	
Other	Glass coverslip 22x32mm	Mariendfeld	Cat # 0101112	
Other	Glass slide	Sail brand	Cat # 7105	
Software	GraphPad Prism9.0	GraphPad		Statistical analysis
Chemical	Lactic acid	Sigma-Aldrich®	Cat # PHR 1215	

compound, drug				
Chemical compound, drug	Linoleic acid	Sigma-Aldrich®	Cat # L1626	
Chemical compound, drug	Lumefantrine	Sigma-Aldrich®	Cat # 1370746	
Chemical compound, drug	Mefloquine	Sigma-Aldrich®	Cat # 1379059	
Chemical compound, drug	Methanol	Merck	Cat#1.06009.2500	
Chemical compound, drug	Piperaquine	Sigma-Aldrich®	Cat # C7874	
Chemical compound, drug	Quinine	Sigma-Aldrich®	Cat # 69311	
Chemical compound, drug	RPMI 1640 medium	HyClone™	Cat # SH30255.01	
Chemical compound, drug	Triton™ X-100	Sigma-Aldrich®	Cat # T8787	
